# Biportal endoscopic laminectomy vs. open-door laminoplasty for 2–3 levels of cervical myelopathy: a prospective observational cohort study

**DOI:** 10.3389/fsurg.2026.1835786

**Published:** 2026-07-02

**Authors:** Dongfang Yang, Mei Li, Haibin Zhang, Mengchen Yin, Mingyang Zhu, Weibing Xu

**Affiliations:** 1 The Second Affiliated Hospital of Naval Medical University (Shanghai Changzheng Hospital), Shanghai, China; 2 Department of Neurosurgery, Dalian Municipal Central Hospital affiliated to Dalian University of Technology, Dalian, China; 3 Department of Orthopedic Surgery, The Second Affiliated Hospital of Inner Mongolia Medical University, Hohhot, China; 4 Department of Orthopedic Surgery, Long Hua Hospital, Shanghai, China

**Keywords:** biportal endoscopy, cervical myelopathy, laminectomy, laminoplasty, minimally invasive spine surgery, propensity score matching, unilateral biportal endoscopy

## Abstract

**Background:**

Unilateral biportal endoscopy (UBE) is increasingly used in cervical spine surgery, but comparative data against open-door laminoplasty (OD-L) for cervical myelopathy (CM) remain limited. This prospective observational study compared biportal endoscopic laminectomy (BE-L) with OD-L for multilevel CM, focusing on neurological recovery, perioperative burden, and sagittal alignment.

**Methods:**

From November 2022 to May 2024, 128 patients with 2–3 level CM were enrolled. Surgical approach followed patient preference. Propensity score matching (1:1) based on age, sex, BMI, preoperative mJOA, operated levels, and C2–C7 Cobb angle produced 50 matched pairs. Primary outcome was ΔmJOA at 12 months. Secondary outcomes included NDI, VASneck, operative time, hospital stay, perioperative hemoglobin (Hb) drop, and sagittal alignment parameters. Because cSVA and T1 slope were not included in the original matching model, a post-hoc sensitivity analysis was performed to verify baseline comparability of these variables.

**Results:**

After matching, baseline characteristics were well balanced. Sensitivity analysis confirmed no significant differences between groups in cSVA (23.1±6.9 vs. 24.8±7.0 mm, P=0.224) or T1 slope ($25.0\pm 5.7^{\circ }$ vs. $26.5\pm 6.0^{\circ }$, P=0.203). BE-L was associated with shorter operative time (88.7±13.8 vs. 146.5±18.4 min, P<0.001), lower perioperative Hb drop (0.62±0.35 vs. 1.98±0.72 g/dL, P<0.001), and shorter hospital stay (2.6±1.2 vs. 7.2±1.6 days, P<0.001). At 3 months, ΔmJOA favored BE-L (mean difference 0.70, P=0.015), but this difference fell below the minimal clinically important difference of 2 points and was not maintained at 6 or 12 months, indicating comparable long-term neurological recovery. Early NDI and VASneck scores favored BE-L, converging by 12 months. No significant differences in C2–C7 Cobb angle were observed at any time point. Four transient neurological deficits occurred in the BE-L group during the initial learning curve; no further events occurred after refinement of hydrostatic pressure management.

**Conclusion:**

BE-L achieves 12-month neurological and functional outcomes comparable to those of OD-L, with advantages in early postoperative recovery and shorter hospitalization. No statistically significant difference was detected at the primary endpoint. BE-L represents a viable minimally invasive alternative for carefully selected patients with 2–3 level CM.

## 1 Introduction

Several posterior cervical decompression surgeries have been adopted for treating cervical myelopathy (CM). The underlying pathophysiology of CM primarily involves direct mechanical compression of the spinal cord secondary to degenerative changes—including osteophyte formation, ligamentum flavum hypertrophy, and disc herniation—as well as secondary ischemic injury resulting from compromised microvascular perfusion. These pathological processes trigger downstream cascades including blood-spinal cord barrier disruption and neuronal apoptosis. The lower cervical spine, particularly the C5–C6 and C4–C5 segments, represents the region of greatest mobility and highest mechanical stress, and consequently constitutes the most frequent site of degenerative stenosis. Elderly patients typically exhibit multilevel involvement, most commonly spanning 2–3 segments [1, 2]. Initially, posterior laminectomy alone was a common option to allow the spinal cord to float posteriorly. However, the postoperative incidence of kyphosis was reported to be unacceptably high, reaching up to 21% [3].Laminoplasty was subsequently introduced into clinical practice as a motion-preserving gold standard for multi-level CM, particularly in patients with preserved cervical lordosis. Nevertheless, concerns persist regarding postoperative axial neck pain, loss of range of motion, and delayed hinge-side closure following open-door laminoplasty (OD-L). Moreover, the necessity of supplemental internal fixation following cervical laminectomy remains debated. Recent large-scale comparative studies have questioned the routine use of instrumentation following laminectomy [4–6], with emerging evidence suggesting a potential trend favoring laminectomy compared to laminoplasty [7, 8]. Notably, recent large-sample studies have demonstrated favorable surgical outcomes of standalone laminectomy for CM [9–11]. A comparative study of 717 patients comparing laminectomy alone vs. laminectomy with fusion for degenerative cervical myelopathy demonstrated that laminectomy alone constitutes a viable option for a carefully selected patient population, particularly those with preoperative physiological lordosis in whom decompression is limited to ≤3 segments [4]. These studies suggest that the development of postoperative cervical kyphosis is more strongly associated with disruption of the posterior musculoligamentous complex (PMLC) than with the laminectomy procedure itself. Recently, a growing number of surgeons have been exploring the value of preserving the PMLC during open posterior cervical surgery [12, 13]. In parallel, a novel minimally invasive technique called unilateral biportal endoscopy (UBE) has gained popularity in the field of spine surgery. This technique employs an arthroscope-like design with independent visualization and instrumentation portals. Under continuous saline irrigation and a magnified visual field, UBE can accomplish surgical decompression comparable to open surgery through a minimally invasive corridor. Recent studies have validated the feasibility and safety of UBE for cervical pathology, demonstrating favorable clinical outcomes for CM treated with UBE-assisted laminectomy [14–17]. However, clinical investigations evaluating purely endoscopic cervical laminectomy remain relatively scarce in the current literature. The extent of preservation of posterior cervical stabilizing structures critically influences postoperative cervical kyphosis and axial symptoms. The minimally invasive endoscopic advantages of UBE enable the performance of laminectomy with minimal disruption to the cervical PMLC. Currently, no comparative studies exist evaluating the surgical outcomes between standalone UBE laminectomy (BE-L) and conventional open-door laminoplasty (OD-L) for CM. To address this gap, we conducted a prospective observational cohort study comparing clinical outcomes and perioperative parameters between BE-L and OD-L for cervical myelopathy.

## 2 Material and methods

### 2.1 Study design and setting

**Figure 1:**
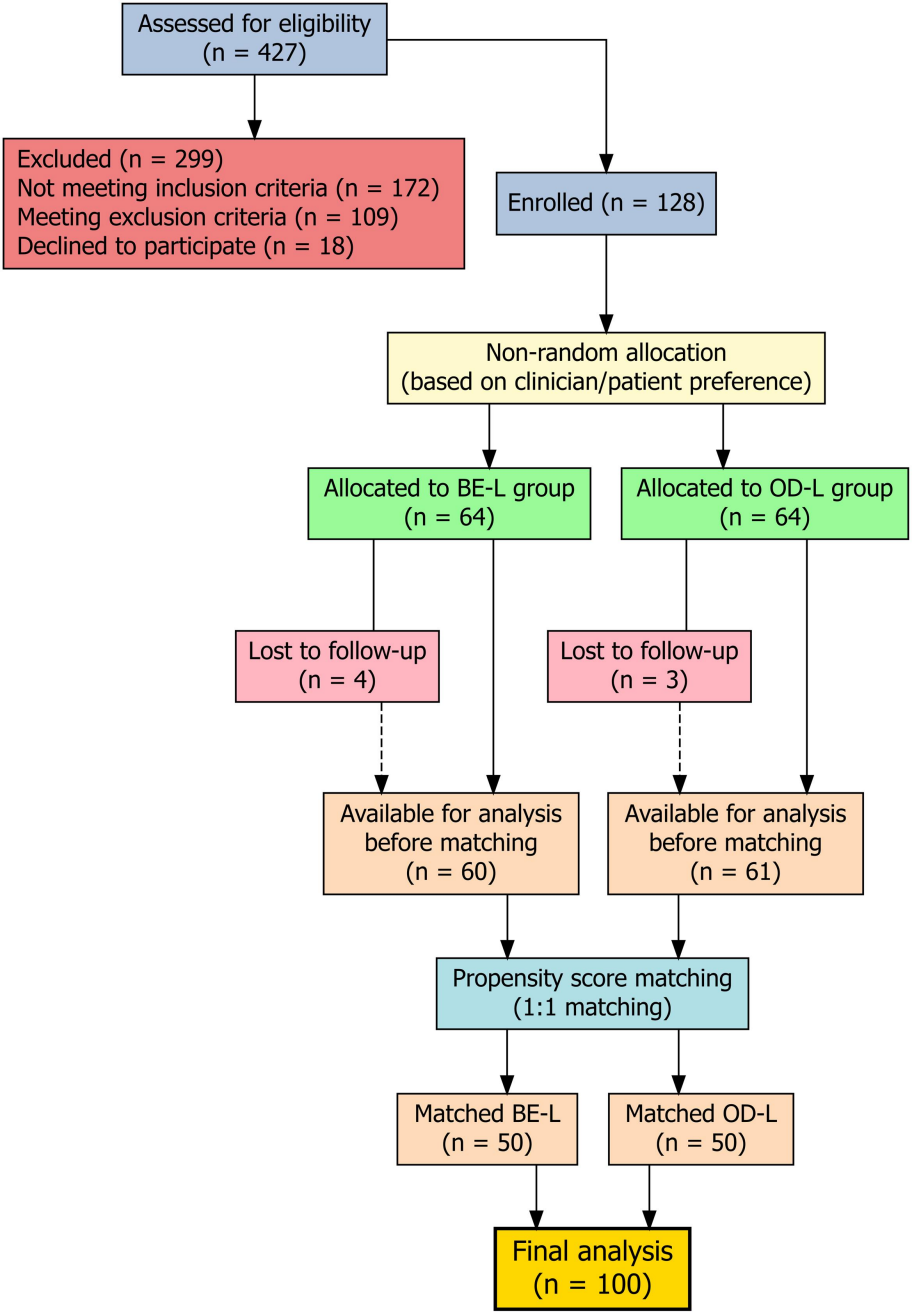
CONSORT flow diagram of the study population and propensity score matching process.

This prospective, observational cohort study was conducted between November 2022 and May 2024 to compare the outcomes of OD-L and BE-L in a real-world clinical setting. The enrollment flow chart is shown in Figure 1. The protocol was approved by the Ethics Committee of Dalian Central Hospital Affiliated with Dalian University of Technology (Approval No. YN2022-089-01) and preregistered at the Chinese Clinical Trial Registry (ID: ChiCTR2200065144, 29 October, 2022), with written informed consent specifically confirming patients’ understanding of surgical alternatives and their voluntary participation in nonrandomized group assignment on the basis of personalized clinical decisions. Although this study used a non-randomized design, it is reported in accordance with the CONSORT Extension for Non-Pharmacological Treatments 2017 [18]. This reporting guideline was chosen because it provides detailed guidance for describing complex surgical interventions, care providers, and centers. Modifications were made to items related to randomization to reflect the non-randomized nature of the study.

### 2.2 Considerations for surgical approach selection

The UBE technique offers the advantage of enabling nearly all manipulations and instrument applications of traditional open surgery within an endoscopic setting, particularly for cervical laminectomy. Since these are not two procedures with comparable iatrogenic trauma—one being distinctly minimally invasive—randomizing patients by surgeon assignment without fully informing them of the differences between the two approaches would, in our view, compromise patients’ right to informed consent and autonomous choice. Therefore, we adopted a process in which an assistant not involved in the study presented animated demonstrations of both surgical techniques to patients and explained their respective advantages and disadvantages. Patients then made their own selection. To minimize selection bias, we applied propensity score matching during statistical analysis. We believe this approach better reflects real-world clinical scenarios.

### 2.3 Study population

Consecutive patients presenting to the Department of Spine Surgery at our hospital with clinical and radiographic evidence of cervical myelopathy involving 2 to 3 levels were screened for eligibility. Inclusion criteria were as follows: (1) aged 40–75 years; (2) clinical signs of myelopathy (e.g., hyperreflexia, positive Hoffmann sign, gait disturbance); (3) 2–3 levels of cervical myelopathy; and (4) MRI-confirmed canal diameter ≤10 mm or Pavlov ratio <0.75. Exclusion criteria were as follows: (1) previous cervical surgery; (2) single-level or >3-level cervical myelopathy; (3) cervical metastatic disease or infection; (4) cervical kyphosis with a negative K-line; (5) instability (dynamic x-ray: translation >3 mm or angulation >10${}^{\circ }$); and (6) other contraindications to general anesthesia. A total of 121 patients met the eligibility criteria and were enrolled in the study cohort. The mean preoperative symptom duration was 6.8±2.3 months in the BE-L group and 7.5±2.2 months in the OD-L group. Prior to propensity score matching, the BE-L group comprised 60 patients and the OD-L group comprised 61 patients. After 1:1 propensity score matching based on age, sex, body mass index (BMI), preoperative mJOA score, number of operated levels, and preoperative C2–C7 Cobb angle, a total of 50 matched pairs (100 patients) were included in the final analysis. Baseline demographic and clinical characteristics of the matched population are summarized in Table 1B. Due to the high proportion of missing preoperative full-spine standing radiographs during the early study period, sagittal parameters including T1 slope and cervical sagittal vertical axis (cSVA) were not incorporated into the propensity score matching model.

### 2.4 Surgical technique

**Figure 2:**
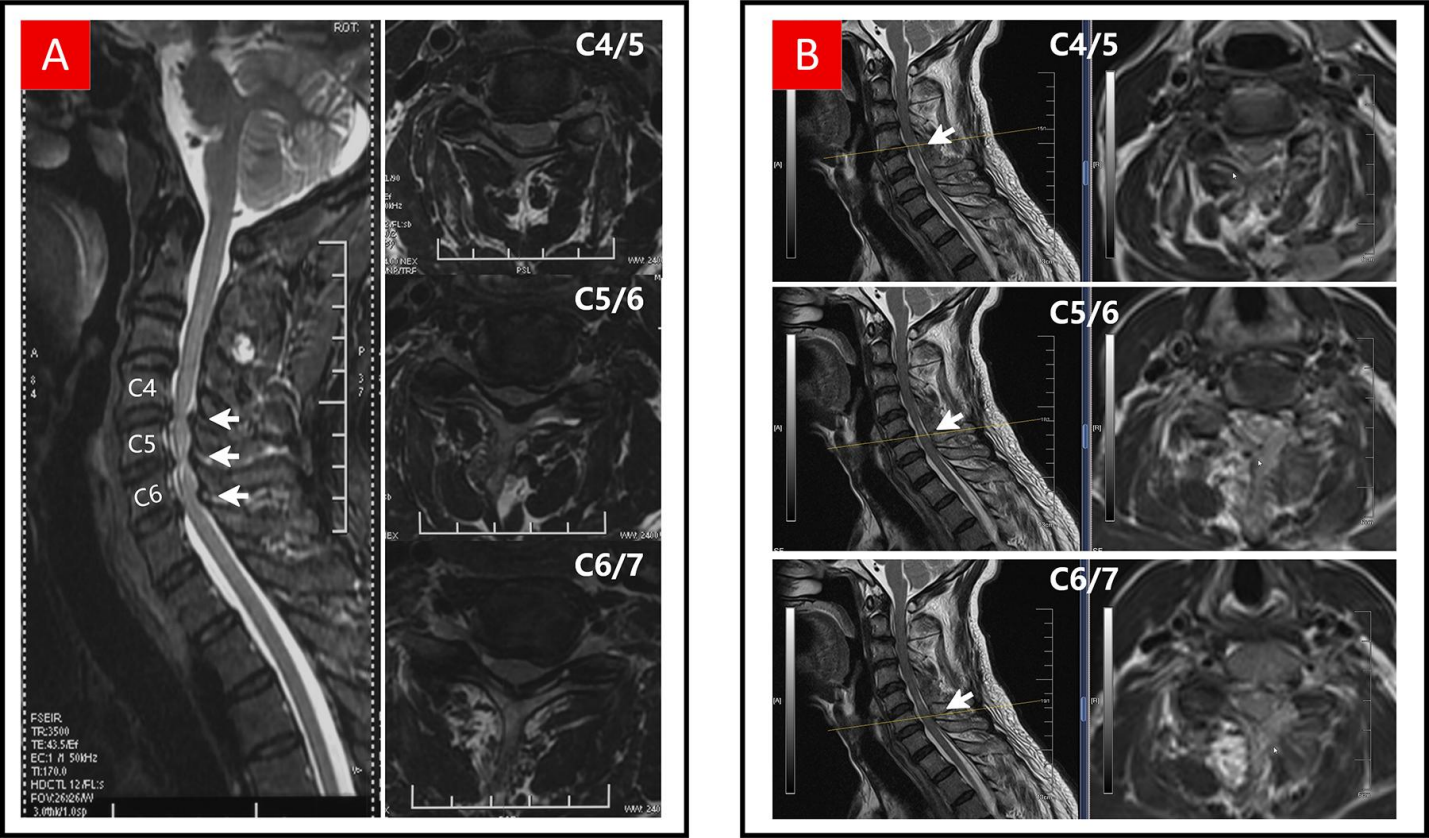
**(A)** Preoperative MRI of the patient showing spinal canal stenosis at C4/5, C5/6, and C6/7 levels. **(B)** Postoperative MRI following UBE posterior cervical decompression.

**Figure 3:**
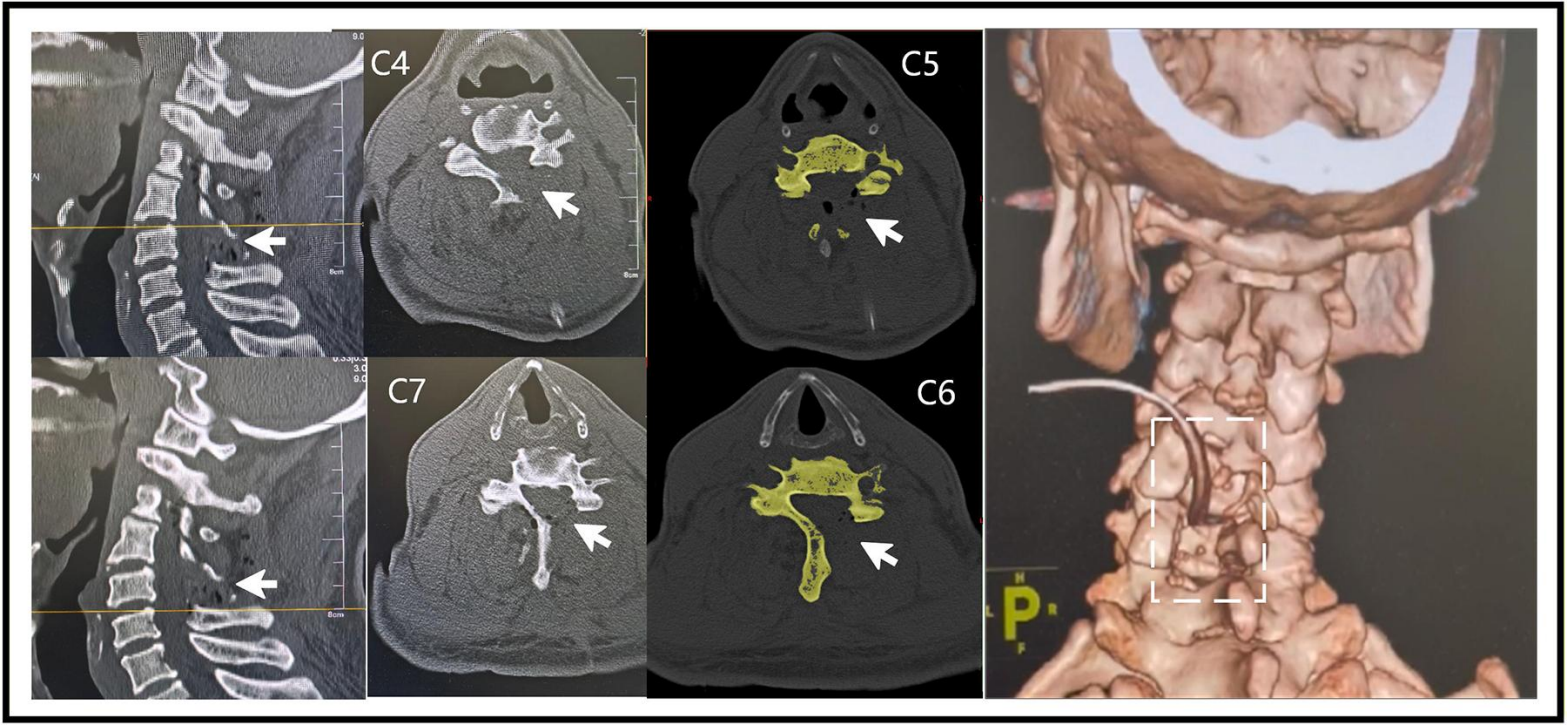
Postoperative CT of the patient demonstrating: Complete laminectomy at C5; Undercutting decompression via hemilaminectomy at C4, C6, and C7.

**Figure 4:**
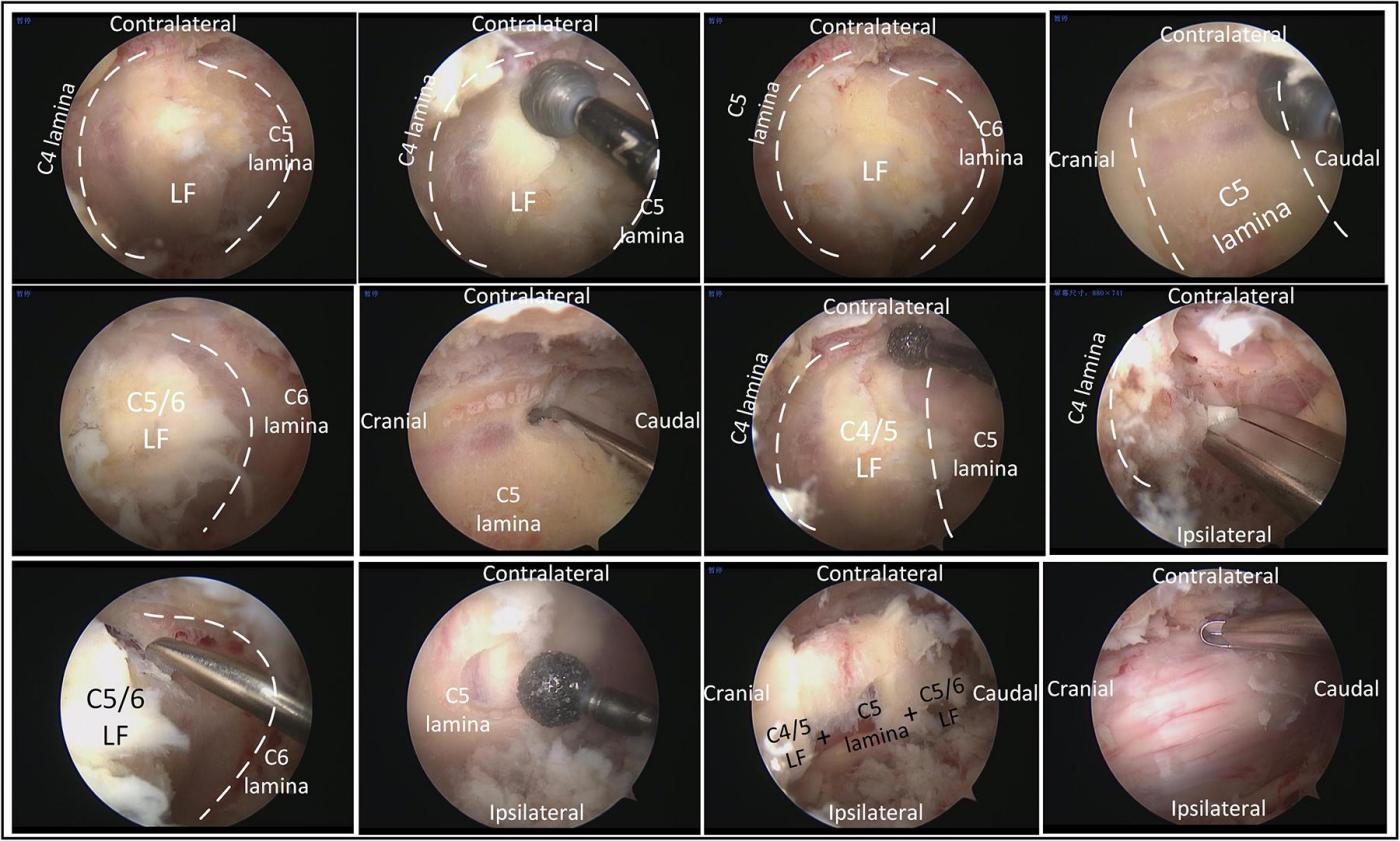
The UBE cervical laminectomy procedure (using a C5 complete laminectomy as an example) allows for the simultaneous resection of the ligamentum flavum at both the C4/5 and C5/6 levels.

Patients in the BE-L group underwent standardized UBE cervical laminectomy (Figures 2, 3, 4). Under general anesthesia, the patients were placed in a prone position. Standard portal establishment was performed, with confirmation of influent and effluent flows. After creating the initial working space, a diamond burr was used to transect the spinous process near its tip. The freed spinous process tip was elevated by water pressure and the tension of the posterior ligamentous complex, which simultaneously expanded the working space and facilitated subsequent laminectomy. Given that the ligamentum flavum covers more than half (52.2% to 70.2%) of the ventral surface of the superior lamina from the cranial (C3) to caudal (C7) levels and does not extend into the foramen, this anatomical knowledge aids in determining the appropriate extent of laminectomy [19, 20]. In cases with concomitant foraminal stenosis, foraminotomy was additionally performed to achieve decompression. After confirming adequate decompression, hemostasis was achieved. A drainage catheter was then inserted to an appropriate depth through the viewing portal, and the skin incisions were closed.

For the patients assigned to the OD-L group, surgery was performed along with the standard posterior cervical laminoplasty procedure [21].Exposure: A midline posterior cervical incision was made, and the paravertebral muscles were dissected subperiosteally to expose the laminae and bilateral facet joints of the involved segments. Creation of the hinge side: On one side, at the medial border of the lamina–facet junction, a high-speed burr was used to remove the outer cortical bone and a portion of the cancellous bone, preserving the inner cortical layer to serve as a rotatable hinge. Creation of the open side: On the contralateral side at the corresponding lamina–facet junction, the lamina was transected completely through its full thickness. Expansion of the spinal canal: The lamina was carefully elevated toward the hinge side, thereby opening the spinal canal in a door-like fashion and achieving significant volumetric expansion until restoration of dural pulsation was visually confirmed. Fixation: The expanded lamina was stabilized either by suture suspension (the original Hirabayashi method involves suturing the spinous process to the facet capsule on the hinge side) or by modern titanium mini-plate fixation to maintain canal expansion and prevent reclosure.

### 2.5 Irrigation fluid and hydrostatic pressure management

Continuous saline irrigation was delivered via an arthroscopic pump system (ConMed Linvatec, USA). The irrigation pressure was strictly maintained between 30 and 40 mmHg, with the saline bag suspended approximately 50–80 cm above the surgical field to avoid excessive epidural pressure [22, 23]. A separate outflow portal was maintained through the working channel to ensure continuous fluid egress and prevent epidural pressure buildup. The surgical team routinely performed an epidural pressure check every 15 min. In the early phase of our experience, four cases of transient neurological deficit occurred, which we attribute to transient outflow obstruction during certain operative maneuvers. Following these events, the protocol was amended to include mandatory intermittent release of the working portal seal. Notably, no further such events were observed after implementation of these refinements.

### 2.6 Perioperative hemoglobin measurement

Hemoglobin (Hb) levels were measured on the day before surgery and on postoperative day 1. The perioperative Hb drop was calculated as the preoperative Hb value minus the postoperative day 1 Hb value, and this parameter was utilized as an objective, irrigation-independent surrogate marker to compare the overall surgical blood loss between the two groups.

### 2.7 Outcome measures

Consecutive adults with symptomatic multi-level CM scheduled for 2–3 level surgery were enrolled. The primary outcome was the paired change in the mJOA score at 12 months, with a minimal clinically important difference (MCID) set at ≥2 points [24]. Secondary outcomes encompassed functional status (Neck Disability Index, NDI), neck/shoulder pain (VASneck), intraoperative parameters (blood loss, operative time), and hospital stay [19]. Baseline demographics, clinical scores (mJOA, NDI, VASneck), and radiographic measures (C2–7 Cobb angle) were collected preoperatively. Patients were followed up at 3 days, 2 weeks, 3 months, 6 months, and 12 months postoperatively for clinical scores, with radiographic assessment repeated at the 3-, 6-, and 12-month visits.

### 2.8 Care providers

In accordance with the CONSORT extension for nonpharmacologic treatments, we report detailed information on the care providers involved in this study. All surgeries in both the BE-L and OD-L groups were performed by the same group of three fellowship-trained spine surgeons at a single tertiary center. The primary surgeon for all cases was a single senior surgeon with extensive experience in both techniques: over 500 OD-L procedures and over 300 BE-L procedures performed before the study period. The remaining two surgeons acted as assistants and had comparable levels of experience across both techniques. This standardization of surgical expertise across groups helps to reduce performance bias and ensures that any observed differences are attributable to the interventions themselves rather than to provider experience.

### 2.9 Sample size calculation

A prospective sample size calculation was performed. Based on detecting a ΔmJOA difference of 1.0 points (SD = 1.5, α=0.05, power = 90%) [20], the initial estimate indicated a need for 48 patients per group. To ensure a sufficiently large patient pool for robust propensity score matching and to account for a 10% loss to follow-up, we set a total enrollment target of at least 120 patients. This target was designed to yield an adequate number of matched pairs for the final analysis, even after accounting for potential exclusions during the matching process.

### 2.10 Radiographic measurements

Preoperative cervical lateral radiographs were obtained with the patient standing in a neutral position, looking straight ahead. To improve visualization of the cervicothoracic junction, patients were instructed to depress their shoulders during image acquisition. Measurements of C2–C7 sagittal vertical axis (cSVA) and T1 slope were performed independently by two spine surgeons blinded to group allocation using the institutional Picture Archiving and Communication System (PACS). The cSVA was defined as the horizontal distance between the posterosuperior corner of the C7 vertebral body and a plumb line dropped from the centroid of the C2 vertebral body. T1 slope was defined as the angle between the superior endplate of T1 and the horizontal reference line.

Among the 100 matched patients, the C7 inferior endplate was clearly discernible in 84 patients (42 in the BE-L group and 42 in the OD-L group), and the T1 superior endplate was adequately visualized in 81 patients (40 in the BE-L group and 41 in the OD-L group). The proportions of non-visualized segments did not differ significantly between the two groups (cSVA: P=1.000; T1 slope: P=0.821). Sensitivity analyses for these parameters were performed on the available cases. Interobserver reliability was assessed using the intraclass correlation coefficient [ICC(2,1) model: two-way random effects, absolute agreement, single measurement], which demonstrated good to excellent agreement for cSVA (ICC = 0.86, 95% CI: 0.80–0.91) and good agreement for T1 slope (ICC = 0.82, 95% CI: 0.74–0.88).

### 2.11 Statistical analysis

Continuous data were presented as mean ± standard deviation or median (interquartile range) based on their distribution, and compared using Student’s t-test or Mann-Whitney U test, as appropriate. Categorical data were expressed as numbers (percentages) and compared using the Chi-squared test or Fisher’s exact test. To address potential selection bias arising from the non-randomized allocation of surgical approaches, we employed propensity score matching (PSM). The propensity score, which represented the probability of a patient undergoing OD-L vs. BE-L, was calculated using a multivariable logistic regression model that included the following pre-operative covariates: age, sex, BMI, involved level, mJOApre., Cobbpre., NDIpre., VASpre., and symptom duration. A 1:1 nearest-neighbor matching algorithm with a caliper width of 0.2 SD was performed to match OD-L patients with BE-L patients. The balance of covariates before and after matching was assessed using standardized differences, with a threshold of ¡0.1 indicating successful balance. Baseline characteristics of both the unmatched and matched cohorts are presented in Tables 1A and 1B, respectively. All primary and secondary outcomes were compared between the two groups in the matched cohort. As a sensitivity analysis to reinforce our findings, we also conducted multivariate logistic or linear regression analyses on the entire unmatched cohort, adjusting for the same set of baseline covariates used in the PSM model. A two-sided p-value of <0.05 was considered statistically significant. All analyses were performed using SPSS version 26.0.

**Table 1A:** Baseline characteristics of patients before propensity score matching.

Variable	BE-L (n=60)	OD-L (n=61)	p-value	Standardized difference
Age (years)	63.2±7.6	60.0±7.2	0.018	0.43
Sex (Male/Female)	22/38	33/28	0.046	−0.28
Involved level (2-level/3-level)	30/30	38/23	0.139	−0.22
BMI (kg/m${}^{2}$)	23.8±2.7	24.9±2.1	0.012	−0.45
mJOApre.	9.3±1.5	9.9±1.6	0.031	−0.39
Cobb anglepre. (${}^{\circ }$)	4.8±4.8	7.3±5.2	0.006	−0.50
NDIpre.	34.5±6.2	37.8±6.5	0.004	−0.52
VASpre.	1.7±1.0	1.5±0.9	0.238	0.21
Symptom duration (months)	6.8±2.3	7.5±2.2	0.085	−0.31
C2–C7 SVA (mm)	22.5±6.8	25.4±7.2	0.026	−0.41
T1 Slope (${}^{\circ }$)	24.3±5.6	27.1±6.1	0.011	−0.48

Data are presented as mean ± standard deviation for continuous variables and as counts for categorical variables. p-values were calculated using independent t-test or chi-square test as appropriate. Standardized difference <0.1 indicates good balance.

**Table 1B:** Baseline characteristics of patients after propensity score matching.

Variable	BE-L (n=50)	OD-L (n=50)	p-value	Standardized difference
Age (years)	61.58±7.52	60.40±7.19	0.424	0.16
Sex (Male/Female)	24/26	27/23	0.548	−0.12
Involved level (2-level/3-level)	28/22	29/21	0.840	−0.04
BMI (kg/m${}^{2}$)	24.23±2.66	24.56±2.10	0.490	−0.14
mJOApre.	9.56±1.50	9.66±1.56	0.745	−0.07
Cobb anglepre. (${}^{\circ }$)	5.34±4.73	6.76±5.20	0.157	−0.29
NDIpre.	35.70±6.18	36.82±6.48	0.379	−0.18
VASpre.	1.66±0.98	1.54±0.91	0.527	0.13
Symptom duration (months)	7.22±2.31	7.12±2.26	0.827	0.04
C2–C7 SVA (mm)	23.1±6.9	24.8±7.0	0.224	−0.24
T1 Slope (${}^{\circ }$)	25.0±5.7	26.5±6.0	0.203	−0.26

Data are presented as mean ± standard deviation for continuous variables and as counts for categorical variables. p-values are derived from your original analysis. Standardized differences were calculated using the formula for continuous and categorical variables.

## 3 Results

### 3.1 Baseline characteristics

Between November 2022 and May 2024, 128 patients were initially recruited. After applying exclusion criteria and accounting for loss to follow-up, 121 patients completed the study (60 in the BE-L group and 61 in the OD-L group prior to matching). The CONSORT flow diagram illustrating patient enrollment, allocation, follow-up, and analysis is presented in Figure 1.

To mitigate potential selection bias and ensure comparability between the groups, propensity score matching (PSM) was performed. This process yielded a well-balanced cohort of 100 patients (50 matched pairs). Table 1A presents the baseline characteristics of the unmatched cohort (n=121). Prior to matching, several between-group differences were observed, including statistically significant disparities in age, sex distribution, BMI, preoperative mJOA score, C2–C7 Cobb angle, NDI, C2–C7 cSVA, and T1 slope (all P<0.05).

After 1:1 PSM based on age, sex, BMI, preoperative mJOA score, number of operated levels, and preoperative C2–C7 Cobb angle, the majority of covariates achieved standardized differences <0.1 (Table 1B). However, several variables—including preoperative C2–C7 Cobb angle (standardized difference =−0.29), NDI (−0.18), C2–C7 SVA (−0.24), and T1 slope (−0.26)—remained above this threshold, indicating imperfect covariate balance for these specific parameters. Although none of these residual differences reached statistical significance in the matched cohort (all P>0.05, Table 1B), they should be acknowledged as potential sources of residual confounding when interpreting the results. In the matched cohort, no statistically significant differences were observed between the BE-L and OD-L groups with respect to age, sex distribution, BMI, number of involved levels, symptom duration, or preoperative functional and radiographic parameters (all P>0.05).

Given that cSVA and T1 slope were not prospectively included as covariates in the PSM model, a post-hoc sensitivity analysis was performed to evaluate the comparability of these sagittal alignment parameters between the matched groups. Measurements were retrospectively obtained from preoperative cervical lateral radiographs. The C7 inferior endplate was clearly visible in 84 of the 100 matched patients, and the T1 superior endplate was adequately visualized in 81 patients, with comparable missingness between groups. As summarized in Table 1B, the mean cSVA in the matched cohort was 23.1±6.9 mm in the BE-L group and 24.8±7.0 mm in the OD-L group (P=0.224). The mean T1 slope was $25.0^{\circ }\pm 5.7^{\circ }$ in the BE-L group and $26.5^{\circ }\pm 6.0^{\circ }$ in the OD-L group (P=0.203). These findings indicate that the two groups were reasonably balanced with respect to cervical sagittal alignment despite these parameters not having been incorporated into the original matching algorithm.

### 3.2 Perioperative outcomes

119 Perioperative data for the matched cohort are summarized in Table 2. The mean operation time was significantly shorter in the BE-L group compared with the OD-L group (88.7±13.8 min vs. 146.5±18.4 min, P<0.001). Although intraoperative estimated blood loss could not be accurately quantified in the BE-L group due to continuous saline irrigation, the mean perioperative hemoglobin drop—calculated as the difference between preoperative and postoperative day 1 hemoglobin levels—was significantly lower in the BE-L group (0.62±0.35 g/dL vs. 1.98±0.72 g/dL, P<0.001). This provides objective evidence of reduced overall surgical burden associated with the minimally invasive endoscopic technique. However, the OD-L group had a substantially longer operative time, which likely resulted in greater intraoperative intravenous fluid administration. The consequent hemodilution may have contributed to the larger Hb drop observed in this group, and the Hb decrement should therefore be interpreted with appropriate caution rather than as a precise measure of surgical blood loss alone. Both hospital stay and time to ambulation were significantly shorter in the BE-L cohort compared with the OD-L cohort (2.6±1.2 days vs. 7.2±1.6 days, P<0.001). These findings are consistent with previously reported perioperative advantages of unilateral biportal endoscopic cervical laminectomy over conventional open posterior procedures, including reduced surgical trauma, lower perioperative hemoglobin decrement, and expedited postoperative recovery [25, 26].

**Table 2:** Perioperative outcomes of the matched cohort.

Variable	BE-L (n=50)	OD-L (n=50)	t	p-value
Operation time (min)	88.74±13.78	146.48±18.41	−17.75	<0.001
Blood loss (ml)	–	269.44±82.85	–	–
Hb drop (g/dL)	0.62±0.35	1.98±0.72	−12.02	<0.001
Hospital stay (d)	2.64±1.19	7.20±1.55	−16.48	<0.001

Data are presented as mean ± standard deviation. Hb drop = preoperative hemoglobin minus postoperative day 1 hemoglobin. p-values were calculated using independent t-test.

### 3.3 Neurological and functional outcomes

Postoperative functional and radiographic outcomes are detailed in Table 3. Regarding the primary outcome, the BE-L group demonstrated a significantly greater improvement in the ΔmJOA score at the 3-month follow-up compared to the OD-L group (mean difference 0.70, 95% CI: 0.14 to 1.26; p=0.015). However, this difference was not maintained at the 6-month (mean difference 0.32, 95% CI: −0.33 to 0.97; p=0.331) or 12-month (mean difference 0.50, 95% CI: −0.06 to 1.06; p=0.077) assessments, indicating comparable long-term neurological recovery between the two techniques. For secondary outcomes, the NDI scores in the BE-L group were significantly lower than those in the OD-L group at both the 3-month (mean difference −5.32, 95% CI: −8.82 to −1.82; p=0.003) and 6-month (mean difference −4.00, 95% CI: −7.12 to −0.88; p=0.013) assessments. By 12 months, the difference in NDI between groups was no longer statistically significant (mean difference −1.30, 95% CI: −3.21 to 0.61; p=0.181).

**Table 3:** Comparison of clinical outcomes between groups.

Outcome	Time	Mean ± SD		Mean difference	t	p
		BE-L	OD-L	(95% CI)		
ΔmJOA	Pre-op	9.56±1.5	9.66±1.56	−0.1 (−0.71, 0.51)	−0.330	0.745
	Δ3 mo	3.4±1.53	2.7±1.3	0.7 (0.14, 1.26)	2.470	0.015
	Δ6 mo	4.38±1.48	4.06±1.78	0.32 (−0.33, 0.97)	0.600	0.331
	Δ12 mo	5.32±1.48	4.82±1.32	0.5 (−0.06, 1.06)	0.180	0.077
NDI	Pre-op	35±6.44	36.02±6.72	−1.02 (−3.63, 1.59)	−0.775	0.44
	3 mo	16.76±8.72	22.08±8.9	−5.32 (−8.82, −1.82)	−3.019	0.003
	6 mo	10.08±7.94	14.08±7.78	−4 (−7.12, −0.88)	−2.544	0.013
	12 mo	4.9±5.29	6.2±4.31	−1.3 (−3.21, 0.61)	−1.347	0.181
CobbC2-C7	Pre-op	5.34±4.73	5.7±4.23	−0.36 (−2.14, 1.42)	−0.401	0.689
	3 mo	5.26±4.58	5.36±4.31	−0.1 (−1.87, 1.67)	−0.112	0.911
	6 mo	5.36±4.42	5.34±4.54	0.02 (−1.76, 1.8)	0.022	0.982
	12 mo	5.38±4.69	5.32±4.47	0.06 (−1.76, 1.88)	0.065	0.948

Data are presented as mean ± standard deviation. ΔmJOA indicates change from baseline in modified Japanese Orthopedic Association score; NDI, Neck Disability Index; Cobb C2–C7, C2–C7 Cobb angle (degrees). Mean difference (95% CI) represents the difference between groups (BE-L minus OD-L) with 95% confidence interval. p-values were calculated using independent t-test. p<0.05 indicates statistically significant difference between groups.

### 3.4 Radiographic outcomes

Analysis of the C2–C7 Cobb angle revealed no statistically significant differences between the BE-L and OD-L groups at any postoperative time point, with all 95% confidence intervals crossing zero (Table 3). At 12 months, the mean difference was $0.06^{\circ }$ (95% CI: $-1.76^{\circ }$ to $1.88^{\circ }$, p=0.948), indicating that both surgical procedures had comparable effects on cervical sagittal alignment within the first postoperative year. As shown in Table 4, analysis of neck/shoulder pain, as measured by the VASneck, revealed significant intergroup differences during the early to mid-term postoperative period. The BE-L group demonstrated significantly lower pain scores than the OD-L group at 3 days (mean difference −1.00, 95% CI: −1.45 to −0.55; p<0.001), 2 weeks (mean difference −0.76, 95% CI: −1.21 to −0.31; p=0.001), 3 months (mean difference −0.82, 95% CI: −1.31 to −0.33; p=0.001), and 6 months (mean difference −0.54, 95% CI: −0.93 to −0.15; p=0.007) postoperatively. Notably, by the 12-month follow-up, the difference in VASneck between the two groups was no longer statistically significant (mean difference −0.12, 95% CI: −0.41 to 0.17; p=0.417).

**Table 4:** Perioperative and follow-up neck/shoulder pain VAS scores.

Outcome	Time	Mean ± SD		Mean difference	t	p
		BE-L	OD-L	(95% CI)		
VASneck	Pre-op.	1.68±0.98	1.54±0.99	0.14 (−0.25, 0.53)	0.710	0.479
	3 d	4.14±0.97	5.14±1.28	−1 (−1.45, −0.55)	−4.409	<0.001
	2 w	2.56±1.1	3.32±1.15	−0.76 (−1.21, −0.31)	−3.389	0.001
	3 mo	1.58±1.13	2.4±1.33	−0.82 (−1.31, −0.33)	−3.334	0.001
	6 mo	1.04±0.88	1.58±1.07	−0.54 (−0.93, −0.15)	−2.755	0.007
	12 mo	0.78±0.62	0.9±0.84	−0.12 (−0.41, 0.17)	−0.815	0.417

Data are presented as mean ± standard deviation. VASneck indicates neck/shoulder pain visual analog scale score. Mean difference (95% CI) represents the difference between groups (BE-L minus OD-L) with 95% confidence interval. p-values were calculated using independent t-test. p<0.05 indicates statistically significant difference between groups.

### 3.5 Neck and shoulder pain

As shown in Table 4, analysis of neck/shoulder pain, as measured by the VASneck, revealed significant intergroup differences during the early to mid-term postoperative period. The BE-L group demonstrated significantly lower pain scores than the OD-L group at 3 days, 2 weeks, 3 months, and 6 months postoperatively (all p<0.05). Notably, by the 12-month follow-up, the difference in VASneck between the two groups was no longer statistically significant (0.78±0.62 vs. 0.90±0.84, p=0.417).

### 3.6 Complications

In the BE-L group, we observed 4 cases of postoperative upper limb numbness and decreased muscle strength. After treatment with intravenous methylprednisolone and mannitol for 3 days, symptoms completely resolved within one month in 3 patients. In the remaining patient, symptoms improved, but residual numbness persisted, with bilateral upper limb muscle strength graded at 4/5. This was considered potentially related to the hydrostatic pressure generated by continuous saline irrigation during surgery. In the OD-L group, 3 patients presented symptoms similar to those of C5 nerve palsy. These patients received methylprednisolone and gradually recovered within 3 months, with no long-term residual symptoms. Additionally, 6 cases of superficial surgical site infection were observed (2 in BE-L, 4 in OD-L); all of these cases were resolved with antibiotic treatment, and no deep infections occurred. No instances of dural tear or epidural hematoma were observed.

## 4 Discussion

The relative clinical merits of standalone cervical laminectomy vs. cervical laminoplasty for the treatment of CM have long been a subject of debate. Recently, however, several large-sample, long-term follow-up studies have demonstrated that standalone laminectomy performed via the conventional open approach yields long-term clinical outcomes that are non-inferior to those of laminoplasty [4, 9–11]. In this evolving context, UBE-assisted cervical laminectomy offers the potential to further reduce the surgical trauma associated with traditional open laminectomy and may theoretically provide superior preservation of the posterior cervical stabilizing structures. To date, several studies have reported favorable clinical outcomes of UBE techniques in the treatment of cervical radiculopathy [16, 27]; however, the application of UBE-assisted laminectomy for CM—and comparative investigations in particular—remains limited.

Cervical laminectomy has long been associated with post-operative kyphosis and instability. However, recent large-scale comparative studies have shown no significant differences in clinical outcomes following cervical laminectomy with supplemental internal fixation, while the fusion group was associated with longer surgical segments and higher procedural costs [4, 28]. On the other hand, multiple studies have indicated that axial symptoms and kyphosis persist, complications strongly linked to the extensive muscle dissection and disruption of the PMLC [12, 13, 29, 30]. The successful application of UBE in thoracic and lumbar surgery has spurred its exploration in the cervical spine. The BE-L technique theoretically minimizes the risk of post-operative kyphosis by maximizing the preservation of the PMLC, potentially making a standalone laminectomy viable without fusion. Clinical validation of this approach could significantly reduce surgical trauma and better preserve cervical range of motion for multilevel CM patients. While initial studiesreport successful BE-L applications, no prospective trials have yet directly compared its outcomes against the standard OD-L technique [14, 15, 17, 31].

### 4.1 Interpretation of primary and secondary outcomes

This prospective observational cohort study compared the outcomes of BE-L and OD-L over a 12-month period. Regarding the primary outcome, the change in mJOA score (ΔmJOA) demonstrated a statistically significant difference favoring the BE-L group at the 3-month follow-up. However, it is crucial to note that the magnitude of this early difference (0.70 points) fell well below the established MCID of 2 points for the mJOA scale. Furthermore, this intergroup difference was not maintained at the 6- and 12-month assessments, at which point no statistically significant difference was detected between the two techniques, indicating comparable long-term neurological recovery. Therefore, while BE-L may offer a transient, subclinical advantage in early functional trajectory, the 12-month primary endpoint confirms that BE-L yields equivalent neurological outcomes to the conventional OD-L approach. We hypothesize that the observed early trend may be attributed to reduced intraoperative manipulation of neural structures and less postoperative intraspinal hemorrhage associated with the BE-L procedure. Secondary outcomes revealed a distinct pattern. The BE-L group demonstrated advantages in early post-operative recovery, evidenced by better NDI and VAS-neck scores at the 3-month mark. This is likely attributable to the minimally invasive nature of BE-L, which causes less soft tissue injury compared to the extensive muscular dissection required in OD-L. By the 6-month follow-up, the difference in NDI, while still measurable, was no longer statistically significant, and by 12 months, most functional scores converged. These findings indicate that the BE-L technique is associated with reduced severity and shorter duration of axial symptoms in the early phases of recovery, though both groups achieved similar levels of pain control and disability in the long term. It is also important to consider the potential influence of self-selection bias on the observed outcomes. In this study, the surgical approach was determined by patient preference after viewing animated demonstrations of both techniques and receiving explanations of their respective advantages and disadvantages. Patients who elected to undergo the novel, minimally invasive BE-L procedure may have had higher baseline functional expectations, greater motivation for rapid recovery, or a stronger preference for avoiding extensive surgical dissection. These psychosocial characteristics are not captured by the variables included in the propensity score model and may contribute, at least in part, to the better early postoperative pain and functional scores observed in the BE-L group. While PSM effectively balanced measured covariates such as age, sex, and preoperative mJOA score, the inherent self-selection mechanism represents a source of residual confounding that cannot be fully adjusted for statistically. This consideration further reinforces the need for cautious interpretation of the between-group differences, particularly during the early postoperative period.

### 4.2 Radiographic outcomes and sagittal alignment

Radiographically, the intergroup comparison of the C2–C7 Cobb angle did not reveal any statistically significant differences between the BE-L and OD-L groups at any follow-up time point, with all confidence intervals crossing zero (Table 3). At 12 months, the mean difference was only $0.06^{\circ }$ (95% CI: $-1.76^{\circ }$ to $1.88^{\circ }$). However, it must be emphasized that the 12-month follow-up in this study is insufficient to adequately address the key long-term concern in this patient population—namely, delayed postoperative kyphosis. The development of post-laminectomy kyphosis can occur gradually over several years, and the stable alignment observed during the first postoperative year does not constitute evidence that either technique provides superior preservation of sagittal alignment over the long term. The theoretical biomechanical advantage of preserving the posterior musculoligamentous complex with BE-L remains just that—theoretical—and cannot be substantiated by the current data. Extended radiographic surveillance beyond 24 months is necessary to determine whether the BE-L approach translates into clinically meaningful differences in sagittal alignment over time.

### 4.3 Technical nuances and anatomical considerations of cervical UBE

**Figure 5:**
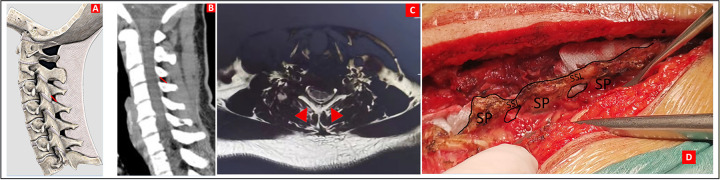
**(A–B)** The absence of ISL; **(C)** Cervical MRI image: fatty space under the cervical paravertebral muscles (red triangles); **(D)** The absence of the ISL was also observed in the cervicothoracic surgery (C6–T2). SP, spinous process; SSL, supraspinous ligament.

On the basis of our technical experience, when UBE techniques applied in the thoracic/lumbar spine are compared with open laminectomy, BE-L presents unique technical nuances. First, at the interlaminar window level, the cervical anatomy differs fundamentally from that of the thoracolumbar region. Unlike the thoracolumbar spine, we observed no substantial “superficial layer of the ligamentum flavum” analogous to that typically encountered in lumbar UBE procedures. Anatomical studies have confirmed that the so-called “superficial layer” in the lumbar spine actually represents an extension of the interspinous ligament, whereas the “deep layer” constitutes the true ligamentum flavum [32]. During BE-L procedures, we consistently noted the absence of this so-called “superficial layer” (i.e., interspinous ligament extension). Instead, after soft tissue clearance and establishment of the initial working portal, we directly access the deep layer of the ligamentum flavum without encountering the interspinous ligament (ISL). This observation prompted further investigation, and our literature review revealed that there was no definitive ISL within the cervical segments (C2–C6) [33–35]. Instead, only a thin, translucent septum was identified as the interspinous connection, which is believed to be an extension and replacement of the ligamentum nuchae and does not provide direct bone-to-bone connections. A potential space filled with adipose tissue exists between the ventral surface of the multifidus muscle and the dorsal surface of the lamina. This space communicates bilaterally through the region where the interspinous ligament is absent—a configuration similarly observed at the upper thoracic level [36], as shown in Figure 5. We have designated this anatomical interval the “Subnuchal Safety Space”, which serves as the anatomical basis for UBE total laminectomy. Thus, the “nuchal ligament–ISL–paraspinal muscle complex” constitutes the core posterior stabilizer of the cervical spine. Cusick et al. proposed that complete facet joint resection risks spinal instability [37], but preservation of posterior tension band structures can restore stability. A biomechanical study confirmed that with intact posterior ligaments, resecting 50% of the facet joint caused no significant changes in cervical stability or mobility [38]. This anatomical characteristic significantly reduces the number of operative steps in cervical BE-L and serves as the foundational anatomical basis for successful BE-L execution. From a safety standpoint, it is important to acknowledge that four patients in the BE-L group (8.0%) experienced transient postoperative neurological deficits. Although all four cases resolved completely within 48 h with conservative management and no permanent sequelae, a complication rate of 8%—even for transient events—is clinically meaningful and should not be dismissed. All four events occurred exclusively within the first 15 cases of the series, and no further neurological events were observed in the subsequent 35 cases after refinement of the hydrostatic pressure management protocol. This temporal clustering strongly suggests that these complications were related to the learning curve and are preventable with meticulous attention to irrigation pressure control and outflow management. Nevertheless, surgeons who are in the early phase of adopting cervical UBE should be aware of this risk and may benefit from proctorship, cadaveric training, or a staged progression from lumbar to cervical procedures before performing these operations independently.

### 4.4 Clinical implications and future directions

Based on the results of this prospective observational study, BE-L demonstrates feasibility and effectiveness as a surgical option for 2–3 level cervical myelopathy. Our findings indicate that BE-L yields neurological and functional outcomes comparable to those of OD-L at 12 months, with no statistically significant differences detected at the primary endpoint, while being associated with reduced early postoperative pain, shorter operative time, and shorter hospital stay. During the 12-month follow-up period, no instances of cervical instability or accelerated kyphosis were documented in the BE-L cohort. These outcomes support the role of BE-L as a potential alternative to established posterior techniques, particularly in contexts where minimizing soft tissue disruption and expediting recovery are prioritized. Furthermore, this study reinforces the hypothetical significance of preserving posterior stabilizing elements in cervical procedures. Future refinements in surgical technique will focus on developing tailored hybrid approaches that strategically combine laminectomy, hemilaminectomy, foraminotomy, and ligamentum flavum resection. These patient-specific decompression strategies represent a promising pathway for enhancing therapeutic efficacy and surgical precision.

## 5 Limitations

This study has several limitations. First, as surgical allocation was based on patient preference, this observational study is subject to residual confounding despite PSM; several matched variables had standardized differences exceeding 0.1, and unmeasured factors—including patient expectations and attitudes toward minimally invasive surgery—may have influenced early outcomes. Second, the 12-month follow-up is insufficient to assess delayed kyphosis or adjacent segment degeneration. Third, the single-center design limits generalizability. Fourth, the perioperative Hb drop is confounded by differential intraoperative fluid administration owing to longer operative times in the OD-L group. Fifth, the four transient neurological deficits in the BE-L group occurred during the learning curve and resolved completely after protocol refinement. Finally, the sample size calculation was based on a 1.0-point ΔmJOA difference, whereas the established MCID is 2 points; the study may therefore be underpowered to exclude clinically meaningful differences between procedures. Future prospective multicenter studies with extended follow-up are warranted.

## 6 Conclusion

This prospective observational cohort study demonstrates that biportal endoscopic laminectomy (BE-L) yields 12-month neurological and functional outcomes comparable to those of open-door laminoplasty (OD-L) in patients with 2–3 level cervical myelopathy, while offering advantages in early postoperative recovery, reduced pain, shorter operative time, and reduced hospital stay. Radiographic outcomes were comparable between groups throughout follow-up. It should be noted that the study was not designed as a formal non-inferiority trial, and no statistically significant difference was detected at the 12-month primary endpoint. BE-L represents a viable minimally invasive alternative for selected patients with multilevel CM. Future research with longer follow-up is essential to confirm its long-term durability and determine whether the observed early advantages translate into sustained benefits.

## Data Availability

The raw data supporting the conclusions of this article cannot be made publicly available due to patient privacy restrictions. Data will be shared by the corresponding author upon reasonable request.
